# Effects of anesthetic method on inflammatory response in patients with Parkinson’s disease: a randomized controlled study

**DOI:** 10.1186/s12871-020-01112-9

**Published:** 2020-08-01

**Authors:** Won Jung Hwang, Min A. Joo, Jin Joo

**Affiliations:** Department of Anesthesiology and Pain Medicine, Seoul St. Mary’s Hospital, College of Medicine, The Catholic University of Korea, 222 Banpodaero, Seocho-gu, Seoul, 06591 South Korea

**Keywords:** Parkinson’s disease, Inhalational anesthetic, Total intravenous anesthesia (TIVA), Inflammatory response

## Abstract

**Background:**

The pathogenesis of Parkinson’s disease (PD) involves degeneration of dopaminergic neurons, which is influenced by innate and adaptive immunity. IL-17 is a characteristic cytokine secreted by Th17 cells, which acts as a powerful stimulator of neutrophil migration and infiltration and promotes the secretion of inflammatory cytokines. General anesthesia and surgical stress induce immune and inflammatory responses that activate the immunosuppressive mechanism in the perioperative period. The present study investigated changes in levels of inflammatory cytokines, such as IL-17, IL-1β, and TNF-α, in patients with PD undergoing general anesthesia with inhalational anesthetics or TIVA.

**Methods:**

Adult patients, aged 40–75 years, scheduled for cerebral stimulator implantation were enrolled. Upon arrival at the operating theater, patients were allocated to the inhalational (I) or TIVA (T) group using block randomization. In group I, anesthesia was induced by tracheal intubation 1–2 min after intravenous administration of propofol (1–2 mg/kg) and rocuronium (0.6–1 mg/kg). Thereafter, anesthesia was maintained with 1–2 vol% sevoflurane, 0.01–0.2 μg/kg/min remifentanil, and O_2_/air (FiO_2_ 0.4). In group T, propofol (3–6 μg/mL), remifentanil (2–6 ng/mL), and rocuronium (0.6–1 mg/kg) were administered using target controlled infusion (TCI) for induction of anesthesia. Blood samples were obtained preoperatively (T0), 2 h after induction of anesthesia (T1), and 24 h after surgery (T2). IL-17, IL-1β, and TNF-α levels were evaluated by ELISA.

**Results:**

Serum levels of IL-17 were elevated at T2 in group I compared to group T but the difference was not statistically significant. IL-1β tended to be greater in group I compared to group T, but the differences were not significant. TNF-α was slightly higher at all time points in group T and showed a tendency to increase at T2 in both groups, but this was not statistically significant.

**Conclusions:**

TIVA may be useful for inhibiting neuroinflammation by inhibiting the increase in serum levels of IL-17 24 h after implantation surgery. Serum IL-17 level may be used as a biomarker for PD progression.

**Trial registration:**

Clinical Research Information Service of Korea National Institute of Health (CRIS) Identification number: KCT0002061. Registered 25 October 2019 - Retrospectively registered, https://cris.nih.go.kr/cris/search/search_result_st01.jsp?seq=15125

## Background

Parkinson’s disease (PD) is a progressive central nervous system (CNS) movement disorder, and is the second most common inflammatory neurodegenerative disorder after Alzheimer’s disease [1]. About 2–3% of elderly people above 65 years old have PD [2]. The earliest symptoms are bradykinesia, resting tremor, rigidity, and impairment of balance. These motor symptoms are caused by a decrease in levels of the neurotransmitter dopamine, due to death of dopaminergic neurons in the substantia nigra [3]. This degeneration of dopaminergic neurons is accompanied by inflammatory changes in microglia (innate immunity) and infiltration of T lymphocytes (adaptive immunity) [4].

There have been a number of studies of the relations between PD and immunity, and Th17 cells play an important role in neurodegeneration in experimental models of PD [5]. In patients with PD, Th17 cell effector molecules are upregulated and the immune pathways of Th17 cells are activated. Th17 cells produce cytokines, such as IL-17, IL-21, IL-1β, and TNF-α, resulting in neuronal apoptosis [5, 6]. IL-17 is a characteristic cytokine secreted by Th17 cells that acts as a powerful stimulator of neutrophil migration and infiltration. It promotes the secretion of other inflammatory cytokines, such as IL-1, TNF-α, and IL-6, by microglial cells [7]. These cytokines lead to neuronal cell death, resulting in neurodegeneration.

General anesthesia and surgical stress induce immune and inflammatory responses that activate the immunosuppressive mechanism in the perioperative period [8]. Unlike inhalation anesthetics, propofol shows antioxidant activity that protects cells and tissues from toxic free radicals [9, 10]. There has been a great deal of research on the relations between general anesthesia and immunity. Inhalational anesthetics have a greater immunosuppressive effect than total intravenous anesthesia (TIVA) using propofol. With the aging of the population, increasing numbers of elderly patients with PD are undergoing various types of surgery under general anesthesia [11]. However, there have been no studies on the effects of anesthetic method on inflammatory responses in patients with PD. The present study investigated changes in levels of inflammatory cytokines, such as IL-17, IL-1β, and TNF-α, in patients with PD undergoing general anesthesia with inhalational anesthetics or TIVA.

## Methods

The protocol of this study adheres to CONSORT guidelines.

### Study population and ethical approval

The study protocol was approved by the Institutional Review Board of Seoul St. Mary’s Hospital, The Catholic University of Korea (approval no. KC17RESI0365) and has been registered with the Clinical Research Information Service of Korea National Institute of Health (CRIS, identification number: KCT0002061). Each patient provided written and oral informed consent. Adult patients, aged 40–75 years who were scheduled for deep brain stimulation (DBS) surgery to control symptoms between June 2018 and December 2019 were enrolled in this randomized, prospective study. Patients who had no medical histories and who had hypertension and diabetes without any complications were included. Patients with myocardial infarction or coronary artery disease, patients with lung diseases, such as asthma or chronic obstructive pulmonary disease (COPD), with AST/ALT greater than normal, and with a medical history of hypersensitivity to inhalation anesthetics or propofol were excluded.

### Anesthetic management

Patients were not allowed to eat and drink 8 h before surgery in consideration of slowed gastric emptying time in PD patients [12], except for the sips of water when they had to take their routine PD medication. Upon arrival at the operating theater, patients were allocated to the inhalational (I) or TIVA (T) group using block randomization. Basic monitoring, including ECG, noninvasive blood pressure (NIBP), pulse oximetry, and bispectral index (BIS), was used. Blood was drawn from peripheral blood vessels before induction of general anesthesia (T0). In group I, anesthesia was induced by tracheal intubation 1–2 min after intravenous administration of propofol (1–2 mg/kg), rocuronium (0.6–1 mg/kg) and remifentanil (0.01–0.2 μg/kg/min). Thereafter, anesthesia was maintained with 1–2 vol% sevoflurane, 0.01–0.2 μg/kg/min remifentanil, and O_2_/air (FiO_2_ 0.4). In group T, propofol (3–6 μg/mL), remifentanil (2–6 ng/mL) using target controlled infusion (TCI), and rocuronium (0.6–1 mg/kg) were administered for induction of anesthesia. After tracheal intubation, anesthesia was maintained with propofol (2–4 μg/mL), remifentanil (2–4 ng/mL), O_2_/air (FiO_2_ 0.4). In both groups, the depth of anesthesia was adjusted to the anesthetic dose with a BIS of 40–60. Blood pressure and pulse were maintained at around 20% of the respective baseline. At the end of surgery, anesthetics were discontinued and sugammadex was used to reverse the muscle relaxant effect. At this time, blood was taken from the peripheral blood vessels (T1). Extubation was carried out when the patient’s breathing had returned sufficiently, and then the patient was moved to the recovery room. Blood was taken from peripheral blood vessels 24 h after surgery (T2). Patients discharged from hospital at postoperative 2 day.

### Cytokine analyses

Blood samples were centrifuged at 1500 rpm for 10 min at room temperature. Serum was removed and stored in 200 μL aliquots at − 80 °C until assays were performed. Serum was dispensed onto coated ELISA plates. Levels of cytokines in the serum samples were determined by ELISA using the following kits in accordance with the manufacturer’s instructions: IL-1β, sensitivity: 6.5 pg/mL (ab46052; Abcam, Cambridge, MA); TNF-α, sensitivity: 4.32 pg/mL (181,421; Abcam); IL-17, sensitivity: < 10 pg/mL (100,556; Abcam). ELISA plates were analyzed using a microtiter plate reader (BioTek Instruments, Inc., Winooski, VT) at 450 nm after stopping the reaction.

### Data collection and statistical analyses

For sample size calculation, IL-17 levels described in a previous animal study were used [12]. With a 10 pg/dL difference in mean value between groups and a standard deviation of 8, the sample size required at a significance level of 5% (two-sided α = 0.05) and a power of 80% (1 – β = 0.8) was 15 patients per group, taking a 10% dropout rate into consideration. All data were analyzed using SPSS (ver. 18.0; SPSS, Inc., Chicago, IL). Demographic data were compared using the χ2 test and t test as appropriate. Repeated-measures ANOVA was performed to compare the cytokines between the groups, with group and time point as independent variables, after confirming the normality of the distribution with the Shapiro–Wilk test (*p* > 0.05). The interaction term was calculated with Bonferroni’s correction for repeated measures. In all analyses, *p* < 0.05 was taken to indicate statistical significance.

## Results

**Fig. 1:**
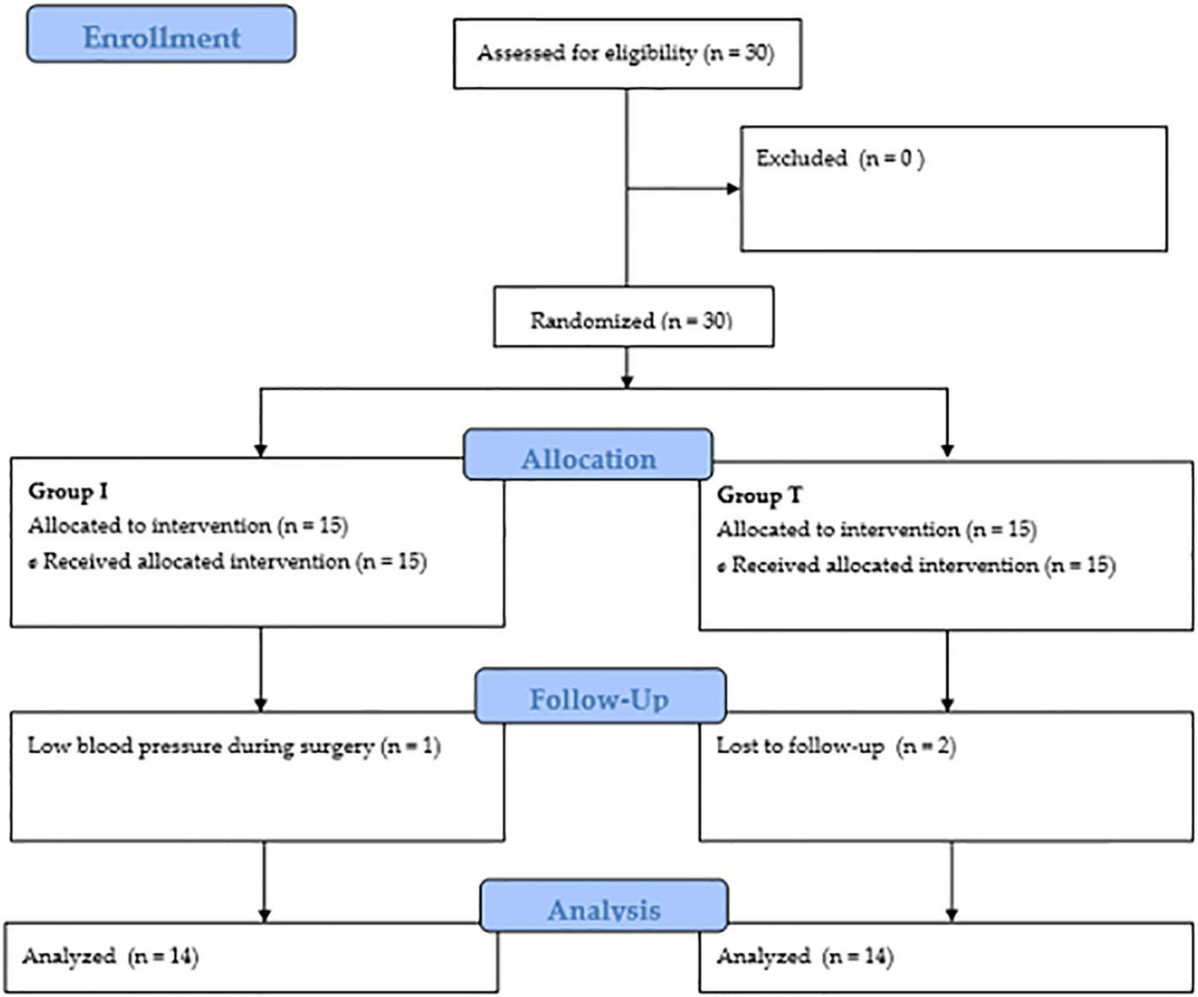
Consort flow diagram

A total of 30 patients were recruited for the study; two were excluded from data analyses (one in each group) due to low blood pressure maintained during surgery and loss to follow-up (Fig. 1). Demographic data and perioperative data are shown in Table 1.

**Table 1:** Patient data

	Group I	Group T	*p*-value
Age (yrs)	71.2 ± 6.4	72.0 ± 6.8	0.987
Sex (M/F)	8 / 6	7 / 7	0.705
Height (cm)	160.6 ± 10.5	161.5 ± 6.7	0.784
Weight (kg)	60.5 ± 12.0	63.4 ± 13.5	0.541
Years after diagnosis of Parkinson’s disease	10.4 ± 6.8	9.5 ± 5.2	0.688
Blood loss (ml)	25.4 ± 16.9	27.9 ± 15.8	0.819
Crystalloid infused (ml)	544.3 ± 305.7	507.9 ± 206.7	0.715
Surgery time (min)	167.5 ± 77.0	177.6 ± 47.5	0.363
Anesthesia time (min)	190.9 ± 34.5	195.0 ± 35.5	0.678

Categorical variables are shown as numbers and other variables are shown as means ± standard deviation

**Fig. 2:**
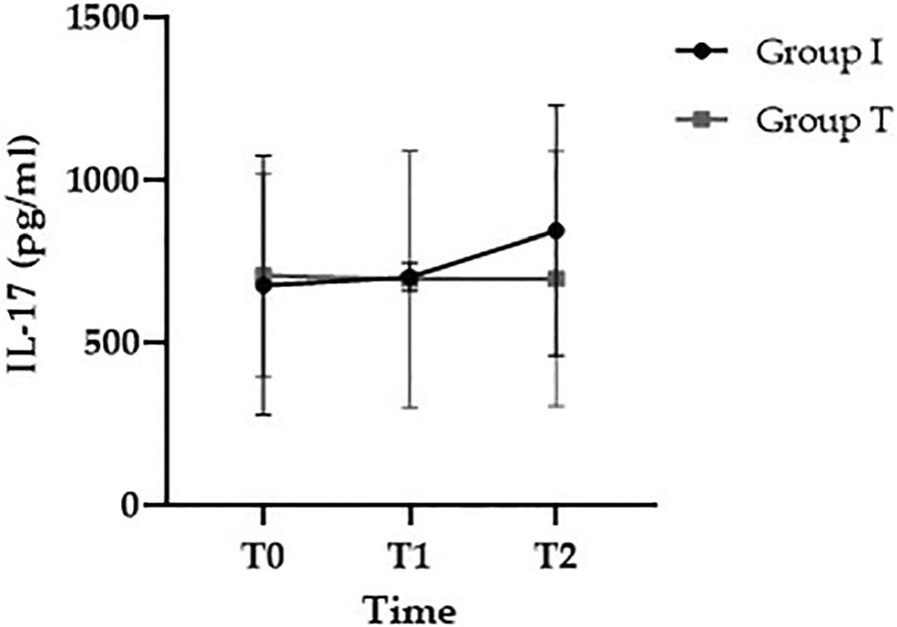
Changes in IL-17. T0, preoperative baseline; T1, 2 h after induction of anesthesia; T2, 24 h after surgery

There were no significant differences in preoperative baseline (T0) IL-17 level between groups I and T (675.6 ± 398.4 vs. 705.7 ± 312.7 pg/mL, respectively; *p* > 0.05). IL-17 did not show any increase at T1 (799.21 ± 423.8 vs. 694.2 ± 395.6 pg/mL, respectively; *p* > 0.05) in either group; it tended to increase at T2 in group I compared to group T (843 ± 384.1 vs. 695.5 ± 391.2 pg/mL, respectively; *p* > 0.05), but the difference was not statistically significant (Fig. 2).

**Fig. 3:**
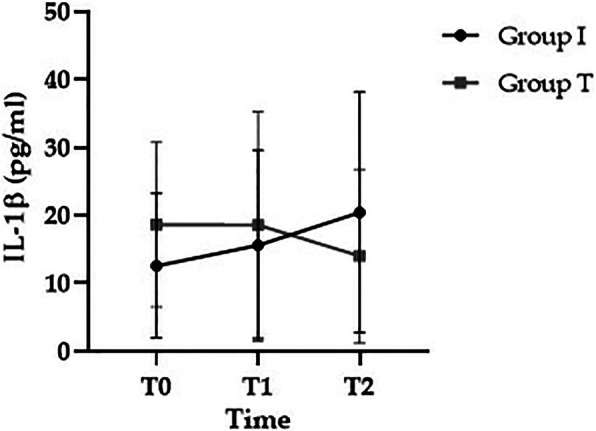
Changes in IL-1β. T0, preoperative baseline; T1, 2 h after induction of anesthesia; T2, 24 h after surgery

**Fig. 4:**
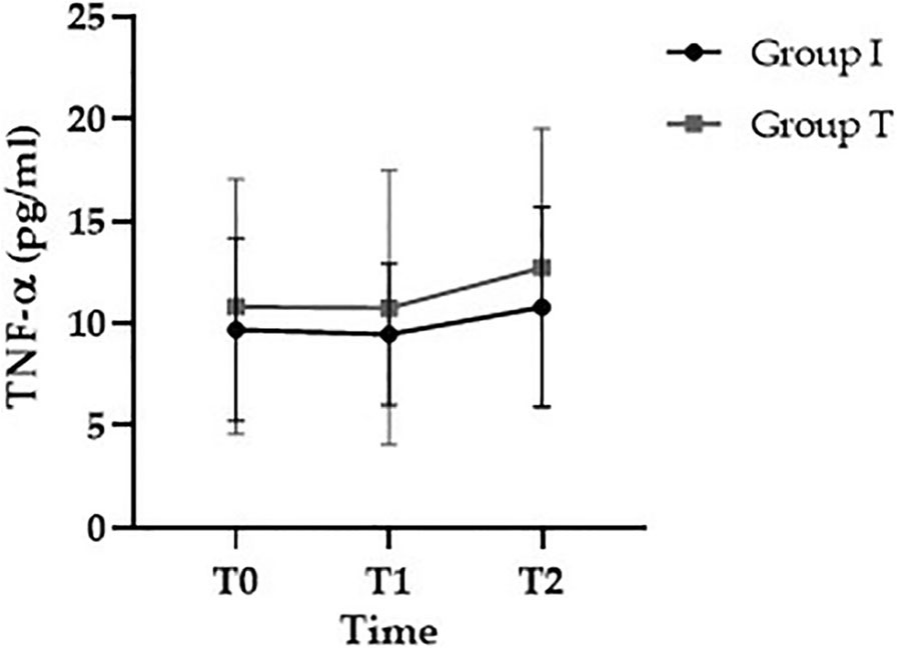
Changes in TNF-α. T0, preoperative baseline; T1, 2 h after induction of anesthesia; T2, 24 h after surgery

There were no significant differences in preoperative baseline (T0) IL-1β level between groups I and T (11.76 ± 9.5 vs. 19.5 ± 11.5 pg/mL, respectively; *p* > 0.05). IL-1β tended to increase in group I compared to group T (15.1 ± 13.0 vs. 16.4 ± 15.1 pg/mL in T1, and 21.2 ± 20.0 vs. 13.2 ± 10.8 pg/mL in T2, respectively; *p* > 0.05), but the differences were not significant (Fig. 3). There were no significant differences in preoperative baseline (T0) TNF-α level between groups I and T (9.9 ± 6.2 vs. 11.9 ± 6.0 pg/mL, respectively; *p* > 0.05). TNF-α showed a tendency to increase at T2 in both groups (9.6 ± 3.8 vs. 11.3 ± 6.4 in T1, and 10.5 ± 4.4 vs. 12.4 ± 8.4 pg/mL in T2, respectively; *p* > 0.05), but this was not statistically significant (Fig. 4).

## Discussion

Various studies have demonstrated that the pathogenesis of PD involves neuroinflammation, which is affected by both innate and adaptive immunity. They have shown that the levels of proinflammatory cytokines are elevated in PD patients [5, 13–16]. Th17 cells are among the most important lymphocytes involved in degeneration of dopaminergic neurons in PD [5]. Th17 cells secrete the proinflammatory cytokine IL-17, which is commonly associated with allergic responses. IL-17 promotes the secretion of other cytokines, such as IL-1β and TNF-α, and plays a pivotal role in the early stages of inflammation. These cytokines bind to receptors on dopaminergic neurons, resulting in apoptosis [4, 6, 17].

To the best of our knowledge, this is the first study to investigate the effects of anesthetic method on inflammatory response in patients with PD. IL-17 at 24 h after surgery tended to increase under inhalational anesthesia, while it was maintained at the preoperative baseline level under TIVA. IL-1β and TNF-α also tended to increase at 24 h after surgery under inhalational anesthesia, but the effect was not significant. Surgical stress and anesthesia induce inflammatory responses by disturbing the balance between pro- and anti- inflammatory cytokines [8], which may result in aggravation of the neuroinflammatory response in PD patients. Previous studies have indicated that TIVA has superior effects in inhibiting inflammatory responses to inhalational anesthetics [9, 18–20]. The influence of different inhalational anesthetics on inflammatory response are still controversial [21–24]. We chose to use sevoflurane, one of the commonly used inhalational anesthetics, because it helps rapid induction and recovery. Shan et al. [25] reported that sevoflurane aggravated the prognosis of PD in a Drosophila model. We attributed the lack of statistical significance in this study to the relatively short duration of DBS surgery and the follow-up due to short hospitalization period. These may not have been sufficient to reveal cytokine changes. A decline of immunity due to surgery and anesthesia is known to occur from roughly 2 h after induction of anesthesia, and the peak of immunosuppression occurs 3 days after surgery [8]. We obtained blood samples 2 h after induction of anesthesia when changes in the immune responses had just begun and 24 h after surgery when changes had not yet reached their peak. Another reason for lack of the statistical significance may be attributed to relatively long period of fasting. Preoperative fasting is one of the stress inducing factors during perioperative periods [26]. Prolonged fasting time may have resulted in the increases in the cytokines in both groups, masking the effect of the different anesthetics on inflammatory response. Guidelines for preoperative fasting period have recently been changed. Meanwhile, gastric emptying time is known to be prolonged in PD patients [12]. Given this, we did not allow the patients to eat and drink from 8 h before surgery, except for the time when they had to take the routine PD medications. However, these results demonstrate the possibility that TIVA have advantages with regard to inhibition of neuroinflammation after surgery in PD patients despite its lack of statistical significance since all the cytokines showed tendency of increase at 24 h after surgery.

The baseline IL-17 level in this study was about 700 pg/mL. Sommer et al. [27] reported that IL-17 levels in PD patients were about 350 pg/mL, while they were below 50 pg/mL in controls. Resting tremor, rigidity, bradykinesia, and impairment of balance are characteristic motor symptoms of PD. Shan et al. [21] reported that impairment of locomotor abilities is aggravated with exposure to sevoflurane in a Drosophila PD model. Tuon et al. [13] reported that IL-17 levels decrease after physical training in an experimental mouse model of PD. Williams-Gray et al. [2] showed that serum levels of cytokines, such as IL-1β, TNF-α, and IL-10, are higher in PD patients than in age-matched non-PD controls; higher TNF-α levels are associated with faster rates of motor decline and higher IL-1β levels are associated with a faster rate of cognitive decline. These studies suggest that IL-17 and TNF-α are closely related to the motor symptoms of PD. Our cohort consisted of patients who had been diagnosed with PD for about 10 years and required DBS surgery to manage motor symptoms that were not controlled by medications. These observations suggest that serum levels of IL-17 increase with the progression of PD. That is, neuroinflammation induced by Th-17 cells causing neuronal cell apoptosis may be an important factor in the progression of PD symptoms. Thus, serum IL-17 may be used as a biomarker for PD progression.

One of the limitations of this study is that we used the results from an experimental animal study for sample size calculation [13] because no similar clinical studies have been reported in the literature. In that study, the authors analyzed the levels of IL-17 in the brain tissue of a mouse model of PD 24 h after the intervention. However, the baseline IL-17 level is different between mice and humans, especially PD patients. Nevertheless, we believe that this study was worthwhile as a cornerstone in that it determined the baseline serum level of IL-17 for use in future human clinical studies on PD. In addition, we did not examine the motor symptoms before and after surgery in relation with cytokine changes. We selected PD patients undergoing brain stimulator implantation, and it would have been difficult to evaluate the motor symptoms before surgery due to the severity of PD and after surgery due to the stimulation. We intend to compare changes in short- and long-term motor symptoms in relation to cytokine changes in a future study.

In conclusion, this study did not demonstrate the superiority of TIVA to sevoflurane in inhibiting neuroinflammation 24 h after DBS surgery in PD patients. However, the larger increase of IL-17 at 24 h after DBS surgery under inhalation study reveals the possibility that TIVA have advantages with regard to inhibition of neuroinflammation after surgery. Serum IL-17 level may be used as a biomarker for PD progression since it was much higher in PD patients with severe motor symptoms. Further clinical trials to investigate the relationships between changes in cytokine levels and motor symptoms are needed.

## Data Availability

The datasets used and/or analyzed during the current study are available from the corresponding author on reasonable request.

## Abbreviations

PD: Parkinson’s disease
CNS: Central nervous system
TIVA: Total intravenous anesthesia
BIS: Bispectral index
DBS: Deep brain stimulation
